# Apoptotic Potential and Chemical Composition of Jordanian Propolis Extract against Different Cancer Cell Lines

**DOI:** 10.4014/jmb.1905.05027

**Published:** 2019-11-22

**Authors:** Nael Abutaha

**Affiliations:** Bio-products Research Chair, Department of Zoology, College of Science, King Saud University, P.O. Box 2455 Riyadh, Saudi Arabia

**Keywords:** Propolis, chromatography, anticancer activity, apoptosis, chemical composition

## Abstract

Propolis is a resinous substance that is collected by *Apis mellifera* from plant sources and is used in traditional medicine. To study the phytochemical constituents and apoptotic potential of Jordanian propolis extract against different cancer cell lines, propolis was extracted using methanol, hexane, and ethyl acetate and was fractionated using chromatographic methods. Cytotoxicity was assessed using MTT and LDH assays. The apoptotic potential was investigated using florescence microscopy, multicaspase assay, Annexin-V and dead cell assay, and cell cycle assay. The phytochemical constituents were analyzed using GC-MS. The methanol extract of propolis exhibited cytotoxic potential against all cell lines tested. The IC_50_ values of the methanol extract were 47.4, 77.8, 91.2, and 145.0 μg/ml for HepG2, LoVo, MDAMB231, and MCF7 cell lines, respectively. The IC_50_ values of the F1 fraction were 31.6 (MDAMB231), 38.9 (HepG2), 36.7 (LoVo) and 75.5 (MCF7) μg/ml. On further purification using thin-layer chromatography, the IC_50_ values of the F1-3 fraction were found to be 84.31(HepG2), 79.2 (MCF7), 70.4 (LoVo), and 68.9 (MDAMB231) μg/ml, respectively. The anticancer potential of the F1 fraction was confirmed through the induction of apoptosis and cell cycle arrest at the G0/G1 phase. The GC-MS analysis of the F1 fraction revealed the presence of 3-methyl-4- isopropylphenol (29.44%) as a major constituent. These findings indicate the potential of propolis extract as a cancer therapy. However, further investigation is required to assess the acute and subacute toxicity of the most active fraction.

## Introduction

Cancer is the second leading cause of mortality worldwide [1]. In 2014, there were an estimated 1,665,540 new cases of cancer and 585,720 cancer-related deaths in the United States [2]. Among the 58,180 cancer cases reported by the Gulf Center for Cancer Registration (GCCR) from 1998 to 2004, 49.06% of cases were males and 50.94%were females. The majority of cases were registered from the kingdom of Saudi Arabia (71.1%), followed by Oman (11.1%), Kuwait (6.8%), Bahrain (5.1%), UAE (3.8%), and Qatar (2.1%) [3]. By 2050, it is expected that 27 million new cancer cases and 17.5 million cancer-related deaths will occur worldwide [4]. Natural products are a source of many compounds with therapeutic effects against cancer. About 47.1% of anticancer drugs are isolated or derived from natural products [5].

Propolis (bee glue) is a plant-derived resinous substance that is metabolized by honeybees (*Apis mellifera*). It has been traditionally been used as a therapeutic agent for millennia. Its biological activities, which range from antioxidant, antimicrobial, anti-inflammatory, antidiabetic, dermatoprotective, anti-allergic, laxative, anticancer and radioprotective [6-7] effects, have been reported in many countries, despite differences in geographical origin and botanical sources [7-8]. These biological activities have also been validated in vivo and in vitro [9]. Propolis has shown promising activity against various cancer cell lines such as those of brain, skin, breast, pancreatic, liver, kidney, prostate, and bladder cancers [7]. The components of propolis that have been shown to have antitumor activities include phenethyl ester, caffeic acid, chrysin, nemorosone, artepillin C, galangin, cardanol, vestitol, neovestitol, isoliquiritigenin, and artepillin [9-10].

In this study, the cytotoxic and apoptotic effects of Jordanian propolis and the phytochemical constituents of its cytotoxic fraction on cancer cell lines were evaluated for the first time.

## Materials and Methods 

### Origin of Propolis

Jordanian propolis (JP) was collected from Jarash. Sample was maintained at −20°C until extraction.

### Solvent Extraction 

Propolis (20 g) was extracted in methanol (500 ml) in a shaking incubator at 150 rpm for 24 h at 25°C. The suspension was centrifuged and then evaporated using a rotovap (Heidolph, Germany) at 45°C. Methanol extract (6 g) was re-extracted in hexane (300 ml) by shaking the mixture at 150 rpm at 25°C for 2 h. The suspension was then decanted and the remaining propolis (solid) was re-extracted in 300 ml of hexane, centrifuged, and the two extracts were pooled together (hexane extract). The remaining residue after hexane extraction was further extracted with ethyl acetate (300 ml) in a shaking incubator similar to hexane. The suspension was then decanted and the remaining propolis was re-extracted in 300 ml of ethyl acetate, centrifuged, and the two extracts were pooled together (ethyl acetate extract). The resulting hexane and ethyl acetate extracts were evaporated, weighed and dissolved in methanol (HPLC-grade) (Fisher Scientific, UK) at a final concentration of 20 mg/ml and stored at -80°C until use.

### Column Chromatography

**Fig. 1:**
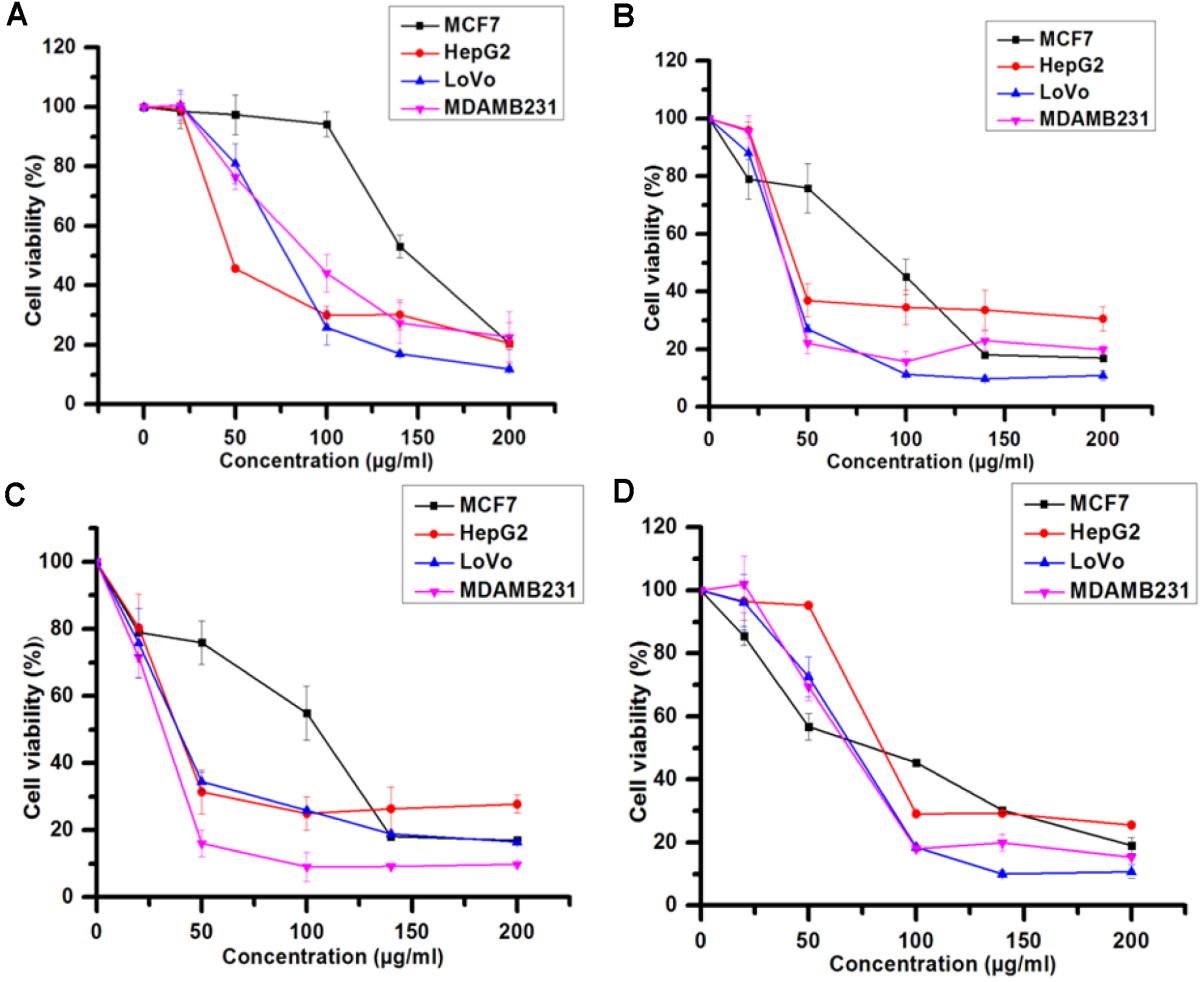
Graph showing cell viability percentage of different cancer cells treated with propolis crude extract and fractions for 48 h. Values are expressed as mean ± std. dev (*n* = 3). A: methanol extract, B: hexane extract, C: F1 fraction and D: F1-3 fraction.

**Fig. 2:**
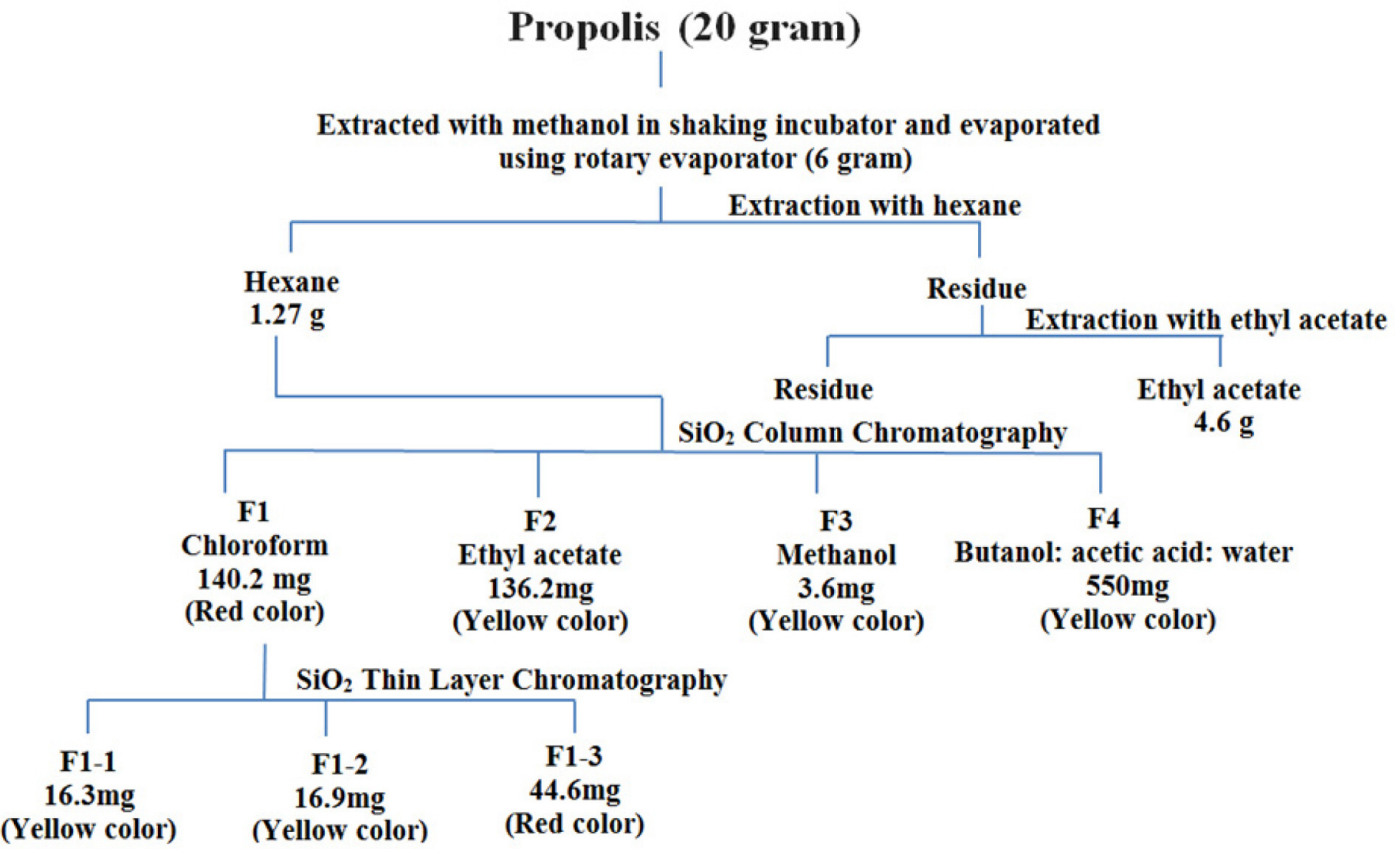
Procedure for the isolation of active fraction from the propolis methanol extract.

**Fig. 3:**
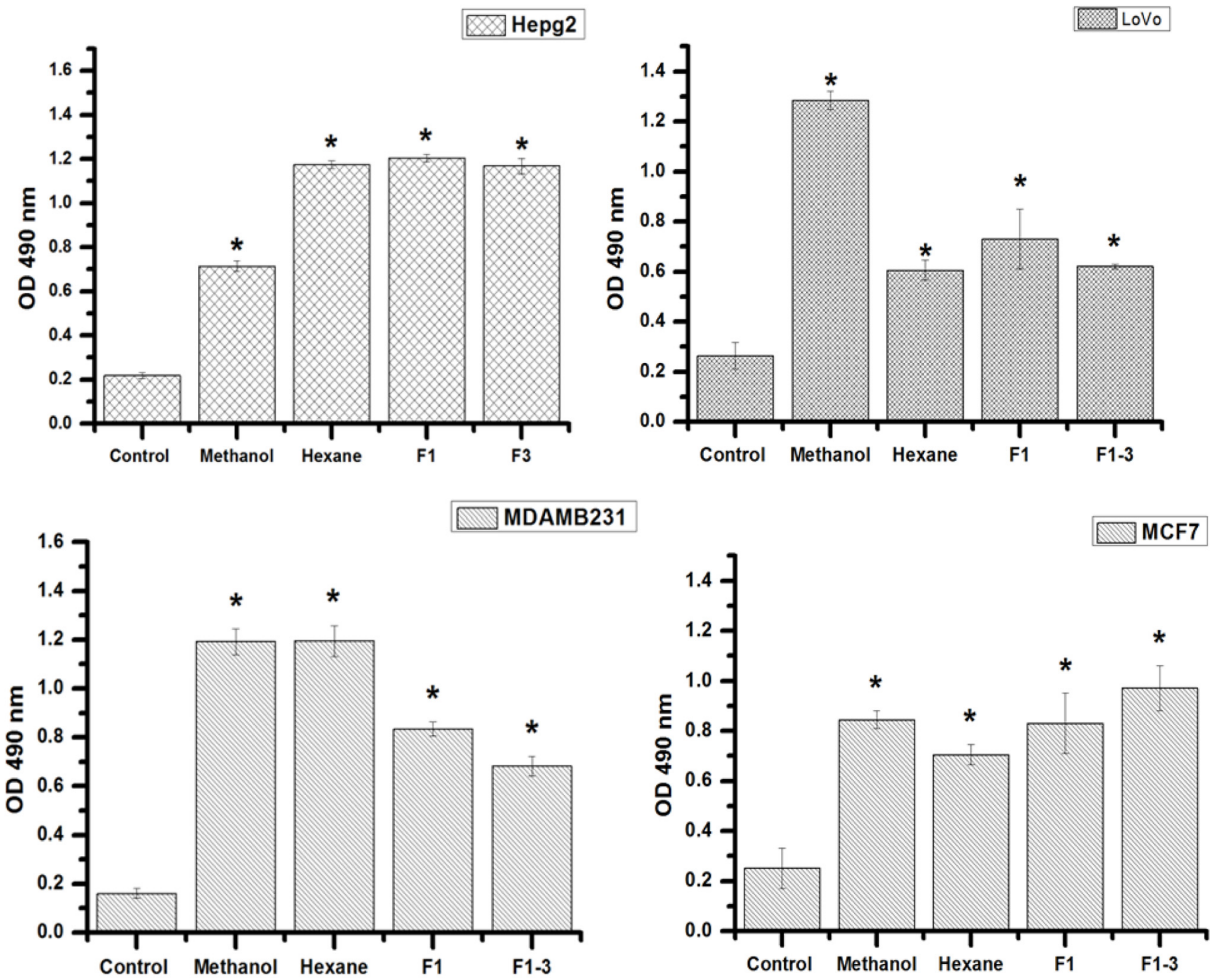
Lactate dehydrogenase (LDH) assay showed the cytotoxicity of the propolis extract and its fractions against LoVo, MDA-MB-231, HepG2, and MCF7 cells. The result revealed significant cytotoxicity at concentrations of 2xLC_50_. The data represent the means of three independent experiments. *p < 0.05 compared with the control group.

**Fig. 4:**
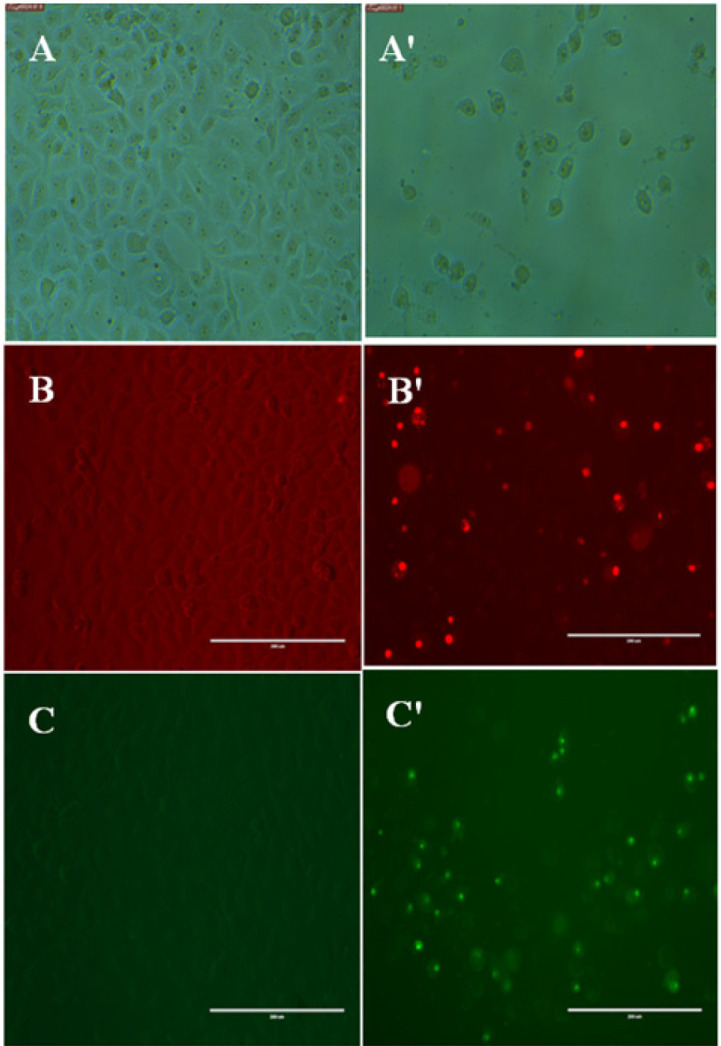
Cell morphology, propidium iodide staining, and Caspase 3/7 assay of human MDAMB231 breast cancer cells treated with 30 μg/ml of the F1 fraction for 48 h; the light and the fluorescence microscopy images were captured at 200× magnification. In light microscopy, control cells (A) displayed normal cellular morphology, whereas treated cells (A') showed shrinkage and cell detachment. In fluorescence imaging of PI stained control cells (**B**) and treated cells (**B**'), the fragmentation of chromatin (abnormal nuclei) and horseshoe-shaped nuclei in the treated cells indicated cell death. Caspase-3/7 activity was more prominent in treated cells (**C**') than in control cells (**C**).

Three grams of silica gel 60 (Riedel-de-Haen, Germany) was mixed with hexane extract, left to dry and then loaded on top of the silica gel-containing column. The column was sequentially eluted with equal volumes (1,000 ml) of chloroform (Fig. 1), ethyl acetate (Fig. 2), methanol (Fig. 3) and butanol: acetic acid: water (4:1:5)(Fig. 4). Pressure was applied to speed the solvent flow rate through the column, and four fractions were collected. The resulting fractions were evaporated using a rotary evaporator at 45°C under vacuum, weighed and dissolved in methanol and stored at −80°C until use.

### Thin-Layer Chromatography (TLC) Analysis

The bioactive fraction (Fig. 1) was further chromatographed using thin-layer chromatography on silica gel 60-(Macherey-Nagel, Germany) coated plates (0.15 mm, 20 × 20 cm) using chloroform as a mobile phase. The TLC plates were dried and then kept in an iodine chamber (1 min). The three visualized bands (F1-1, F1-2 and F1-3) were scraped off and extracted with chloroform. The following formula was used to calculate the R_f_ values:

R_f_ = distance of the spot center from the start point/ distance of the solvent run from the start point.

### Cell Culture

All cell lines were obtained from the German Collection of Cell Cultures (DSMZ, Germany). The selected cell lines were LoVo (human colorectal adenocarcinoma), MCF7 and MDA-MB-231 (human breast cancer), and HepG2 (human hepatocarcinoma). The cells were maintained in Dulbecco’s modified Eagle’s medium (DMEM) (UFC Biotech, Saudi Arabia) supplemented with 10% fetal bovine serum (Gibco, UK), 1% penicillin and streptomycin (Gibco).

### MTT Assay

Cells were seeded in 24-well plates at a density 5 × 10^5^ cells/ml at 37°C and 5% CO_2_. The cells were incubated with increasing concentrations (200-20 μg/ml) of the propolis extracts and fractions diluted in DMEM medium. Cells in DMEM medium treated with 0.01% methanol served as control. After 72 h incubation, the extracts and fractions were tested for viability using the 3-(4, 5-dimethylthiazol-2-yl)-2, 5-diphenyltetrazolium bromide (MTT, Invitrogen). The medium was aspirated, 1 ml of fresh media was added, followed by adding 100 μl/well of MTT solution (5 mg/ml). The plates were incubated for 2 h at 37°C and 5% CO_2_. The formazan crystals formed were solubilized in 0.1% HCl-MeOH (1 ml/well). The absorbance was measured at 570 nm (Thermo Scientific Multiskan, China) and the cell viability % was determined as follows:

Cell viability (%) = (Abs 595 treated cells/ Abs 595 control cells) × 100%.

Results are reported as mean (±SD) resulting from three independent experiments. The IC_50_ values were determined using Origin Software (version 8.5).

### LDH Release Assay

MDA-MB-231 cells were plated into 24-well plates at a cell density of 5 × 10^5^ cells per well, cultured overnight, and treated with propolis as mentioned above at the concentration of 2× (LC_50_). LDH release was examined by LDH cytotoxicity assay kit (Sigma, USA) as instructed in the manual. Absorbance was measured at 450 nm. The experiments were carried out three times.

### Light Microscopy and PI Staining

To determine the effects of propolis extract on the morphology of MDA-MB-231, the cells were cultured in DMEM as mentioned previously. After 48 h, the cells were photographed using a light microscope (Leica, Germany). For fluorescence staining, morphological changes were also observed with PI staining. Treated MDA-MB-231 cells were fixed with ethanol. Then, the cells were stained by adding 1 mg/ml propidium iodide (Invitrogen, USA) for 15 min (37°C) in the dark. Cells were observed under a fluorescence microscope (EVOS, Thermo Fisher Scientific) at 200 × magnification.

### Annexin-V and Dead Cell Assay

Annexin-V & Dead Cell Kit (7-AAD) (Merck Millipore, USA) was used to assess apoptosis using Muse Cell Analyzer (Millipore, USA). Briefly, 5 × 10^5^ cells/ ml were seeded in a 6-well plate for 24 h at 37°C. The MDA-MB231 cells were then incubated with 30 and 60 μg/ml of F1 fraction for 48 h. After incubation, the cells were trypsinized and re-suspended in 1% FBS. Equal volumes of (100 μl) Muse Annexin-V & Dead Cell reagent and cells were pipetted to 1 ml centrifuge tubes, followed incubation at 25°C for 20 min. The assay could differentiate four types of cells: i) nonapoptotic dead cells: Annexin-V (−) and 7-AAD (+), ii) non-apoptotic live cells: Annexin-V (−) and 7-AAD (−), iii) late apoptotic cells: Annexin-V (+) and 7-AAD (+), and iv) early apoptotic cells: Annexin-V (+) and 7-AAD (−).

### Caspase-3/7 Activity Assay

Caspase-3/7 activity was assessed using caspase-3/7 Green Detection Reagent (Invitrogen, CA). MDA-MB-231 cells (5 × 10^5^ cells/well) were seeded in a 4-well plate (Nest, China), incubated for 24 h, and then treated with propolis. After 48 h caspase-3/7 Detection Reagent was added at a final concentration of 10 mM. Cells were observed using a fluorescence microscope. This experiment was repeated three times.

### Multicaspase Assay 

Multicaspase activity percentage was quantified using a Muse MultiCaspase Assay Kit (Merck Millipore, USA). MDA-MB-231 cells (5 × 10^5^ cells/well) were seeded into 6-well plate for 24 h in triplicate and treated with 30 and 60 μg/ml of F1 fraction for 48 h. The Muse Cell Analyzer (Millipore, USA) was used to evaluate multi-caspase activity according to manufacturer’s directions.

### Cell Cycle Assay

This assay was carried out using a Muse cell cycle kit (Merck Millipore). Briefly, MDA-MB-231 cells (5 × 10^5^ cells/well) were incubated in 6-well plate for 24 h at 37°C. The cells were then treated with 30 and 60 μg/ml of F1 fraction. The cells were fixed in ice-cold 70% ethanol for 3 h and then washed with PBS and mixed with 200 μl of Muse cell cycle reagent. After 30 min of incubation at 25°C, the percentage of cells in Go/G1, S and G2/M was analyzed using Muse cell analyzer software (Merck Millipore).

### Gas Chromatography–Mass Spectrometry (GC-MS).

Chemical composition analysis was carried out using a GC-MS (Agilent Technologies, USA) equipped with the NIST library. HP-88 capillary standard column (100 m, ID: 250 μm) of 0.20 μm film thickness was used for the study. The temperature program employed was as follows: the column temperature started at 50oC and was increased to 250oC at 5oC/min. Helium was the carrier gas, the flow rate was 1.0 ml/min, and 2 μl was the injected volume.

### Statistical Analysis

Data were presented as the means ± standard deviation. The analysis was performed using one sample Student’s t-test. Values of *p* < 0.05 were considered as statistically significant.

## Results

The cytotoxicities of the methanol, hexane, and ethyl acetate extracts of propolis and their fractions were assessed against four human cancer cell lines, namely, the estrogen receptor positive (ER+) MCF-7 cell line, the estrogen receptor negative (ER-) MDA-MB-231, HepG2, and LoVo. The viability percentage of the four cancer cell lines upon treatment with the propolis extracts and their fractions was determined after 48 h. The responses of cancer cells to increasing concentrations of propolis extracts and fractions are shown in Fig. 1. The results revealed that the viability of all cell lines decreased in a dose-dependent manner with an increase in the treatment concentration.

The methanol extract exhibited a cytotoxic potential against all the cell lines tested after 48 h of treatment. The IC_50_ values of the methanol extract after 48 h of treatment were 47.41, 77.88, 91.29, and 145 μg/ml for the HepG2, LoVo, MDA-MB-231, and MCF7 cell lines, respectively (Fig. 1). Therefore, this extract was subjected to bioassay-guided fractionation to explore the solubility of its components in different solvents having different polarities (Fig. 2). The hexane soluble fraction showed lower IC_50_ values for the cancer cells compared to the methanol extract, suggesting that the extract could have a promising anti-cancer activity. The IC_50_ values of the hexane extract after a 48 h treatment were 38.7, 38.7, 43.7, and 92.8 μg/ml for MDAMB231, LoVo, HepG2, and MCF7 cell lines, respectively. Upon further fractionation, silica-gel column chromatography (30 mm i.d., 400 mm length) was performed on the hexane extract (1.27 g), yielding 4 sub-fractions (Figs. 1-F4 and 2).

The Fig. 1 fraction showed lower IC_50_ values for the cancer cells compared to the methanol and hexane extracts, indicating that the Fig. 1 fraction could have a potential anti-cancer effect. The IC_50_ values of the Fig. 1 fraction after a 48 h treatment were 31.6, 36.7, 38.9, and 75.5 μg/ml for MDAMB231, LoVo, HepG2, and MCF7 cell lines, respectively. Upon further purification using thin-layer chromatography, the F1-3 fraction showed higher IC_50_ values for the cancer cell lines compared to the F1 fraction, suggesting that these extracts could have a synergistic potential as an anti-cancer agent. Following 48 h treatment with the F1-3 fraction, the IC_50_ values were found to be 79.2, 68.9, 70.48, and 84.41 μg/ml for the MCF7, MDA-MB-231, LoVo, and HepG2 cell lines, respectively (Fig. 1, Table 1).

LDH released into the extracellular environment relates to the degree of cell membrane damage. The LDH release test was used to support the results of the viability assays using 3-(4,5-dimethylthiazol-2-yl)-2,5-diphenyltetrazolium bromide (MTT) following the exposure of cells to the propolis extract. The results shown in Fig. 3 demonstrate that a concentration of 2×LC_50_ of all the fractions of the propolis methanol extract tested significantly increased LDH leakage from LoVo, MCF7, MDA-MB-231, and HepG2 cells, confirming the cytotoxicity of the propolis methanol extract and its fractions.

In the control group, cells were normal, angular, and intact with large vesicular nuclei and prominent nucleoli, and the cells were connected and adhered tightly to the plate. Treated cells started to lose their normal shape, rounded up, detached, and appeared to be vacuolated; their cytoplasm was shrunken and the chromatin was condensed (Figs. 4A and 4A'). The nuclear morphology of MDA-MB-231 cells stained with the PI dye was observed. In the control group, the nuclei of cells showed an even distribution of fluorescence. In the treated group, the numbers of cells decreased and the nuclei appeared fragmented and darker than normal cells, indicating apoptotic cell death (Figs. 4B and 4B'). We next evaluated whether the cytotoxic potential of the Fig. 1 fraction was associated with apoptosis induction. Caspase-3/7 was assayed, and, as expected, propolis treatment caused the activation of caspase 3/7 in MDA-MB-231 cells (Figs. 4C and 4C').

**Fig. 5:**
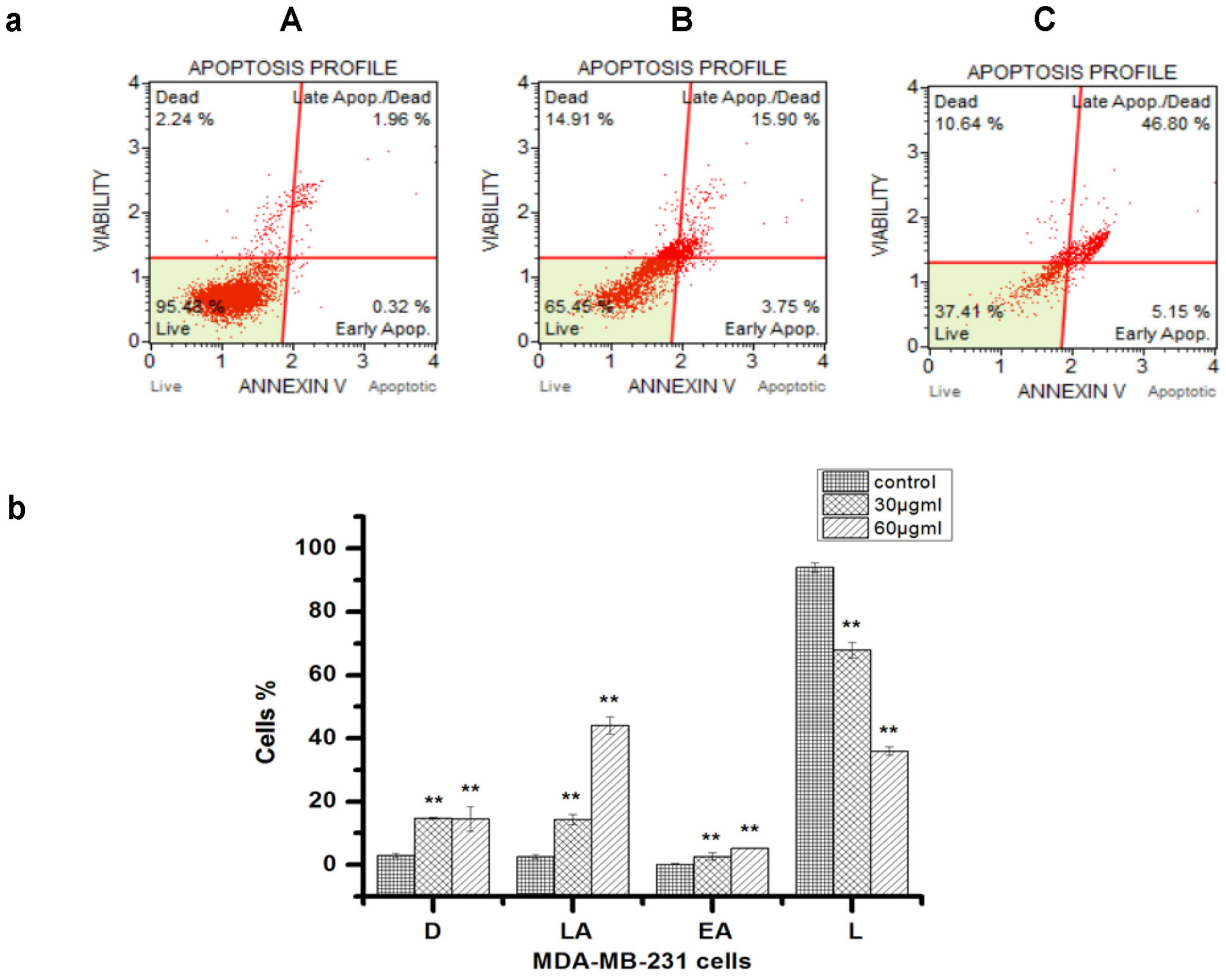
F1 fraction-activated apoptosis in MDA-MB-231 cells. (**a**) Flow cytometry plot for Annexin-V & dead cell analysis of cells treated with different concentrations of the F1 fraction for 24 h. Control group (**A**), 30 μg/ml group (**B**), and 60 μg/ml group (**C**). (**b**) Percentage of apoptotic cells post treatment with 30, and 60 μg/ml of F1 fraction for 48 h. Values were mean ± SD from three independent experiments (*n* = 3). **Indicates significant difference between means and differences were considered significant at *p* < 0.05. VC: vehicle control. L: live cells; EA: early apoptotic cells, LA: late apoptotic cells (cells in late stage of apoptosis or died by apoptotic pathway, AnnexinV-PE(+) and dead cell marker (7-AAD) (+) ); D: dead cells (cells are dead due to necrosis but not via apoptotic pathway, AnnexinV-PE(-) and dead cell marker (+)).

The results indicated that the F1 fraction was cytotoxic for the cancer cell lines tested (Fig. 5). Treatment of the MDA-MB-231 cells with 30 and 60 μg/ml of the F1 fraction of the propolis extract significantly inhibited cell viability to 67.8 ± 2.4 and 36.0 ± 1.5% compared to the corresponding vehicle controls, which had a cell viability of 93.9 ± 1.5. Treatment with 30 and 60 μg/ml also increased the percentage of late apoptotic cells in the MDA-MB-231 cell line, which were 14.3 ± 1.5 and 44.0 ± 2.7%, respectively, in comparison with vehicle controls, which had an apoptotic cell percentage of 2.6 ± 0.6%. The same concentrations also significantly increased the early apoptotic cells to 2.6 ± 1.0% and 5.1 ± 0.02%, respectively, as compared to the vehicle control, which had an apoptotic cell percentage of 0.19 ± 0.1%. These results suggest that F1 fraction of the propolis extract suppresses MDA-MB-231 cell viability through the apoptotic pathway.

**Fig. 6:**
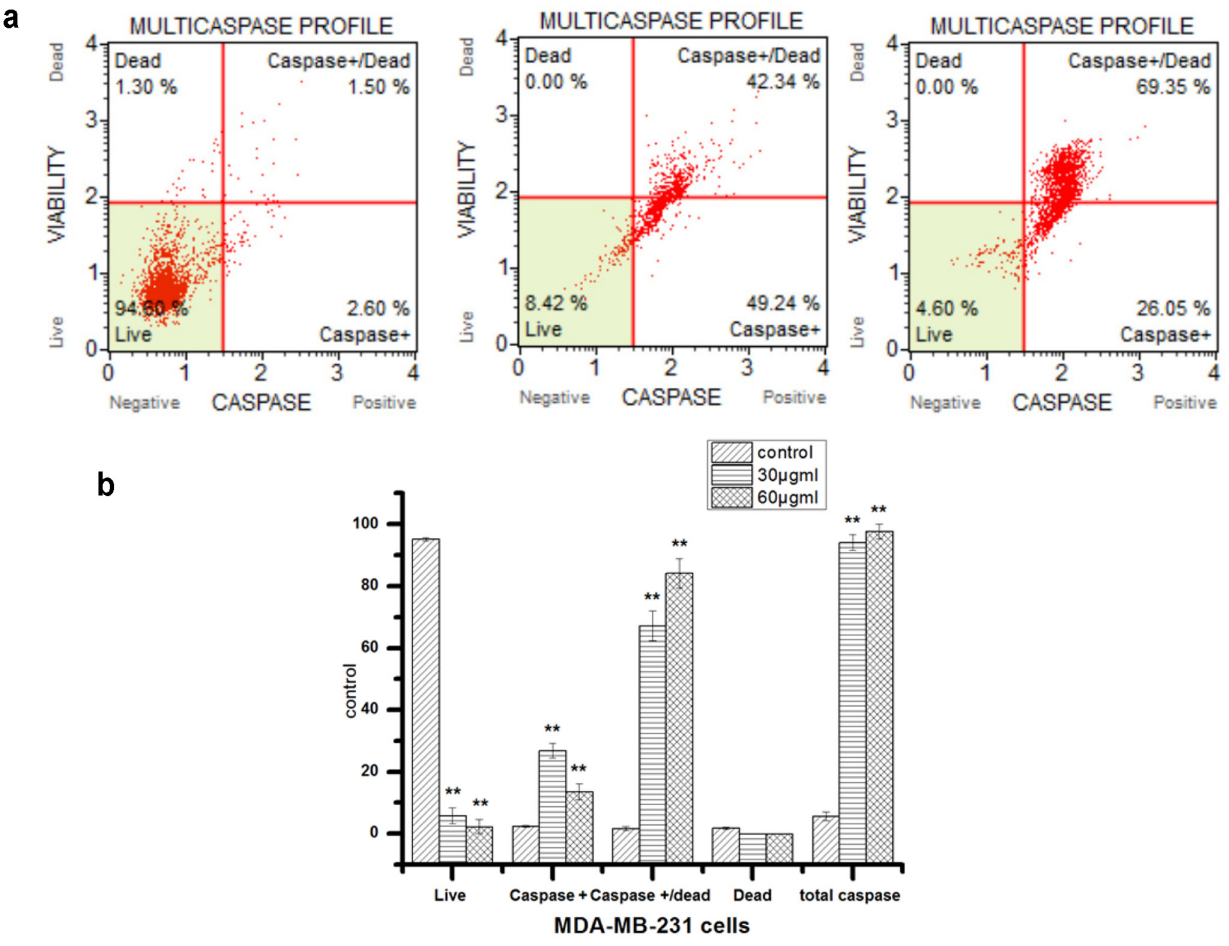
The F1 fraction activates the multicaspase enzyme in MDA-MB-231 cells. (a) Flow cytometry plot for cell viability and multicaspase enzyme analysis of cells treated with the F1 fraction for 48 h. Control group (A), 30 μg/ml group (B), and 60 μg/ml group (C), (b) The bar charts represent the percentages of apoptotic cells post treatment with 30 and 60 μg/ml of propolis extract for 48 h. Values are mean ± SD from three independent experiments (*n* = 3). **Indicates significantly different values, *p* < 0.05. live cells (caspase (-) and dead cell marker (7-AAD) (-)); Caspase+: (caspase (+) and dead cell marker (-)); caspase+/Dead: (caspase (+) and dead cell marker (+)); Dead: cells died through necrosis but not via apoptosis (caspase (-) and dead cell marker (+)).

The results showed that the F1 fraction of propolis extract significantly increased the percentage of MDA-MB-231 cells with induced caspases (Fig. 6). Treatment of MDA-MB-231 cells with the F1 fraction significantly increased the percentage of cells with activated caspases, which were 91.58 ± 2.5% and 95.4 ± 1.8 for the 30 and 60 μg/ml group, respectively, compared to the corresponding vehicle control (7 ± 1.45). This suggests that the F1 fraction of the propolis extract was able to cause caspase-dependent apoptosis in the MDA-MB-231 cells.

**Fig. 7:**
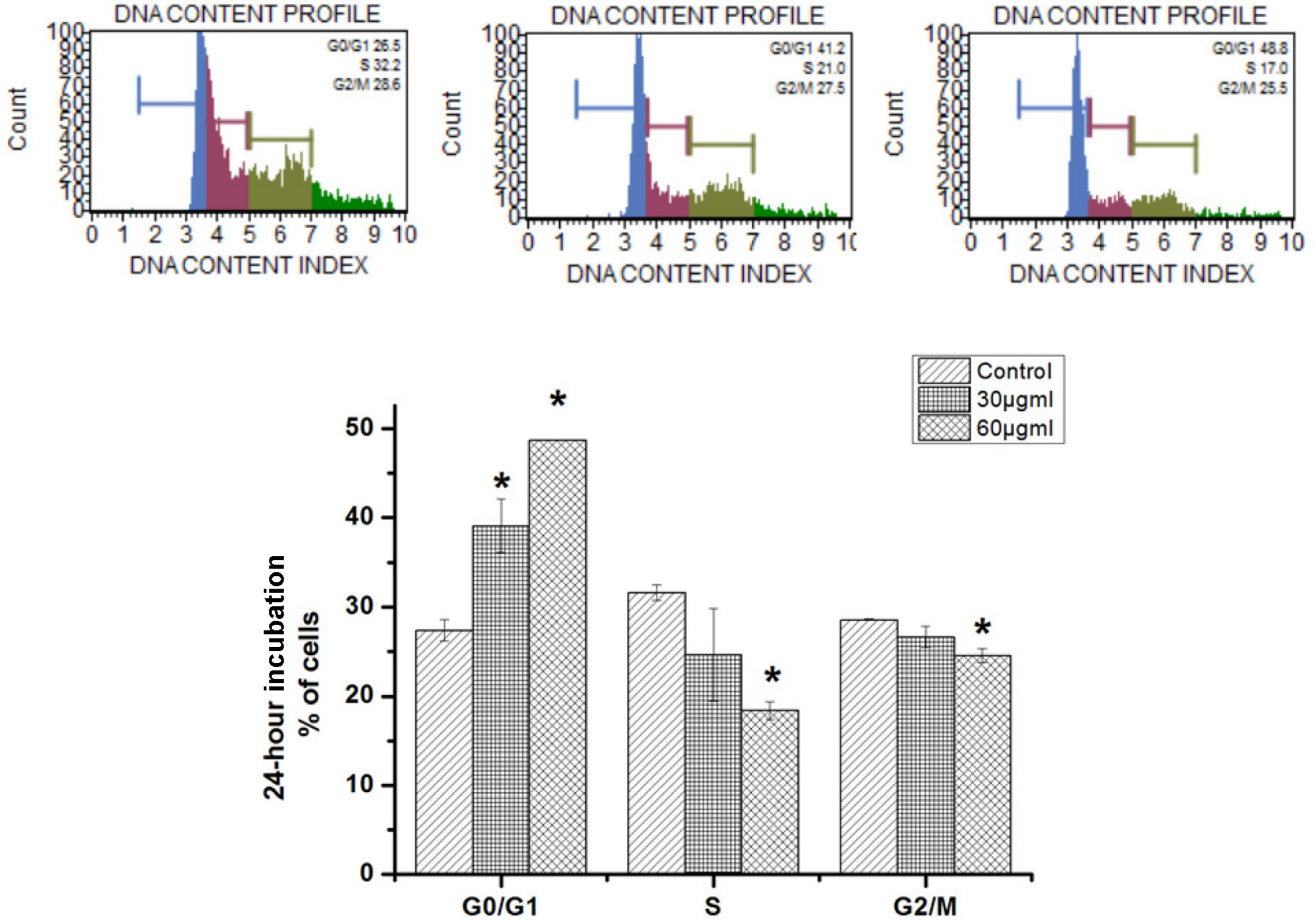
Effects of F1 fraction on the cell cycle of MDA-MB-231 analyzed using the Muse cell analyzer. The cells were treated with 30, and 60 μg/ml for 48 h. Values are mean ± SD from three independent experiments (*n* = 3). *Indicates that the values are significantly different as compared to the untreated control cells, *p* < 0.05.

The effect of treatment with 30 and 60 μg/ml of the F1 fraction for 48 h on MDA-MB-231 cell cycle distribution was assessed using the Muse cell analyzer. As shown in Fig. 7, treatment of MDA-MB-231 cells with F1 fraction at the doses of 30 and 60 μg/ml for 48h showed a significantly higher percentage (39.1 ± 2.9% and 48.7 ± 0%respectively) of cells in the G0/G1 phase compared to the control group (27.3 ± 1.2%, p <0.05), with a corresponding decrease in the cell percentages in the S phase (24 ± 5.1% and 18.4 ± 0.9%, respectively, *p* < 0.05) and G2M phase (26.6.1 ± 1.2% and 24.5 ± 0.7%, respectively) as compared to the control group (31.6 ± 0.8% and 28.5 ± 0.7%, respectively). A more marked arrest of the G0/G1 phase was detected at 60μg/ml (48.7 ± 0.0%) when MDA-MB-231cells were treated for 48h as compared to the control group (19 ± 2.1%, *p* < 0.001) (Fig. 5). These data suggest that the cell death or inhibition of cell proliferation in MDA-MB-231 cells caused by the F1 fraction is related to induction of the G0/G1 arrest.

**Fig. 8:**
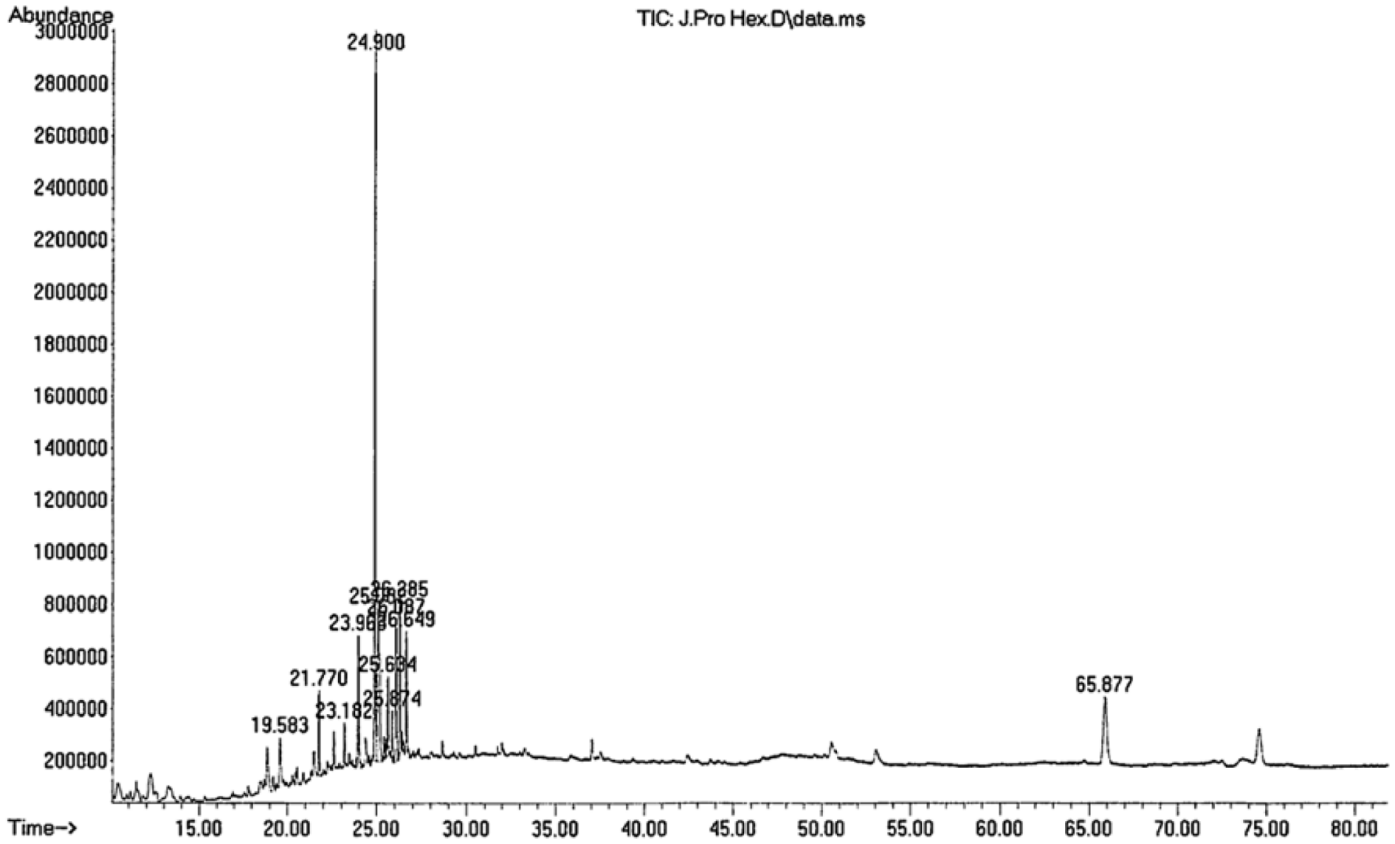
GC-MS of F1 fraction isolated from propolis extract.

The GC-MS analysis of the F1 fraction revealed the presence of 12 compounds. The major chemical constituents were as follows: 3-methyl-4-isopropylphenol (29.44%), naphthalene, 1,2,4a,5,6,8a-hexahydro-4,7-dimethyl-1-(1-methylethyl) (15.42%), 4,8,13-cyclotetradecatriene-1,3-diol, 1,5,9-trimethyl-12-(1-methylethyl)(12.50%) (Fig. 8, Table 2).

## Discussion

Natural products are a valuable source of anticancer drugs [11]. There is much ongoing research on the use of natural products as sources of selective and effective cancer therapies [12, 13]. For the first time, the cytotoxic potential of Jordanian propolis crude extract and its fractions on different cancer cell lines has been studied. Our data demonstrated that propolis treatment resulted in morphological changes, with significant cytotoxic and apoptotic effects on cancer cells lines.

Anti-cancer agents that induce apoptosis are one of the most effective strategies for chemotherapy [14]. Thus, when searching for new anticancer agents, the candidate anticancer agents should demonstrate apoptosis induction in cancer cells [15].

Propolis was investigated for its apoptosis induction ability in MDA-MB-231 cells and the underlying mechanism of action. Apoptosis is characterized by morphological hallmarks, including detachment, cell shrinkage, rounding of cells, membrane blebbing, and condensation of nuclear chromatin [16]. These changes were observed in the MDA-MB-231 cells treated with the F1 fraction (Fig. 4).

One of the important indicators of apoptosis is the translocation of phosphatidylserine, which is detected using Annexin V [ 17]. Our results revealed that apoptosis was increased in a dose-dependent manner when cells were treated with the F1 fraction of the propolis extract. However, apoptosis was activated through the initiator as well as executioner caspases, that is caspases 1 to 9. This is in agreement with reports that confirmed the ability of propolis to initiate apoptosis through both the extrinsic and intrinsic pathways [18-20].

The cytotoxic potential of the F1 fraction for MDA-MB-231 cells was attributable to its potential to induce cell cycle arrest. Interestingly, 30 and 60 μg/ml of propolis significantly arrested the MDA-MB-231 cell cycle in the G0/G1 phase. Collectively, it is evident that the F1 fraction inhibited MDA-MB-231 cell proliferation by inducing G0/G1 phase cell cycle arrest. This is in agreement with the results reported in other studies regarding the effect of propolis extracts on other cancer cell lines [21, 22]. The G0/G1 phase allows cells to trigger apoptotic pathways or repair mechanisms [23]. Thus, the potential of F1 fraction on apoptosis induction of MDA-MB-231 cells was evaluated, and the results showed that treatment of MDA-MB-231 cells with the F1 fraction significantly induced apoptosis. Propolis also induces apoptosis and inhibits cell growth by causing cell cycle S or G2/M phase arrest in different cancer cell types [24-26].

Through the GC-MS analysis of the propolis extract, the presence of 2-naphthalenemethanol, 1, 2, 3, 4, 4a, 5, 6, 7-octahydro-. alpha.,. alpha., 4a, 8-tetramethyl-, (2R-cis)-, which has antibacterial potential against *Staphylococcus aureus* and colibacillus, was revealed. In addition to being used as a food additives propolis is used in cosmetics and in industrial production [27]. Similarly, GC-MS analysis of the propolis extract also revealed the presence of 2-naphthalenemethanol, decahydro-. alpha.,. alpha., 4a-trimethyl-8-methylene-, [2R-(2. alpha., 4a. alpha., 8a. beta.)]-, which is used to treat cough, phlegm, and diuresis [28, 29]. In addition, 3-methyl-4-isopropylphenol, which has been isolated from *Calea urticifolia* and *Plectranthus amboinicus* [29, 30], has also been reported as a bioactive metabolite with antimicrobial, antioxidant, and analgesic activities [17]. Marcucci [30] reported that the cytotoxic potential of propolis against ovarian cancer cell lines was attributable to the naphthalene derivatives in propolis.

This study showed that the F1 fraction of the methanol extract of propolis has cytotoxic potential against different human cancer cell lines. Through bioassay-guided isolation, we identified the F1 fraction as the active fraction responsible for the anti-cancer activity of propolis. Our data suggest that the F1 fraction inhibits the growth of MDA-MB-231 cancer cells through the induction of apoptosis. These findings reveal the potential therapeutic value of the F1 fraction of propolis extract, and further studies in animal tumor models will be important in validating its anti-cancer potential in vivo.

## Figures and Tables

**Table 1:** IC_50_ values of the propolis extract and its fractions for four different cell lines after 48 h of treatment.

Extract	IC_50_ μg/ml			

	HepG2	MCF7	LoVo	MDAMB231
Methanol extract	47.41	145.0	77.88	91.29
Hexane fraction	43.71	92.83	38.7	38.7
F1	38.99	75.5	36.70	31.64
F1-3	84.41	79.2	70.48	68.93

The data represent the means of three independent experiments.

**Table 2:** Components of F1 fraction isolated from propolis using column chromatography as determined by GC-MS.

	Chemical formula	Formula	Molecular weight	Rt (min)	% of total	Bioactivity	Reports from other plants
1	2H-2,4a-Ethanonaphthalene, 1,3,4,5,6,7-hexahydro-2,5,5-trimethyl-	C15H24	204.35	19.58	4.07	-	^11^
2	Propanoic acid, 2-methyl-, 2-phenylethyl ester	C12H16O2	192.25	21.77	3.37	-	^12^
3	Phenethylamine, p-methoxy-α-methyl-,	C10H15NO•ClH	201.69	23.18	1.76	-	^13^
4	Guaiol	C15H26O	222.36	23.96	7.38	Antibacterial, ^14^ insecticidal ^15^, and antitumoral ^16^	^17^
5	3-methyl-4-isopropylphenol	C10H14O	150.21	24.90	29.44	Antimicrobial, antioxidant, and analgesic ^18^	^19^
6	Naphthalene, 1,2,4a,5,6,8a-hexahydro-4,7-dimethyl-1-(1-methylethyl)-	C15H24	204.35	25.08	15.42	-	^20^
7	Naphthalene,1,2,3,4,4a,5,6,8a-octahydro-4a,8-dimethyl-2-(1-methylethenyl)-	C15H24	204.35	25.63	3.86	-	^21^
8	2-Propenoic acid, 3-phenyl-, ethyl ester, (E)-	C11H12O2	176.21	25.87	2.05	-	^21^
9	longipinene	C14H22	190.324	26.08	7.63	Antifeedant and cytotoxic activity ^22^	^23^
10	2-Naphthalenemethanol, 1,2,3,4,4a,5,6,7-octahydro-α,α,4a,8-tetramethyl-, (2R-cis)-	C15H26O	222.36	26.28	7.10	-	^24^
11	2-Naphthalenemethanol, decahydro- alpha.,.alpha.,4a-trimethyl-8-methylene-, [2R-(2.alpha.,4a.alpha.,8a.beta.)]-	C15H26O	222.36	26.64	5.39	-	^24^
12	4,8,13-Cyclotetradecatriene-1,3-diol, 1,5,9-trimethyl-12-(1-methylethyl)-	C20H34O2	306.48	65.87	12.50	-	^25^
